# Timing of Early-Life Stress and the Development of Brain-Related Capacities

**DOI:** 10.3389/fnbeh.2019.00183

**Published:** 2019-08-06

**Authors:** Erin P. Hambrick, Thomas W. Brawner, Bruce D. Perry

**Affiliations:** ^1^The ChildTrauma Academy, Houston, TX, United States; ^2^Lab PANDA, Department of Psychology, University of Missouri—Kansas City, Kansas City, MO, United States; ^3^Center for Research Methods and Data Analysis, University of Kansas, Lawrence, KS, United States; ^4^Department of Psychiatry, Feinberg School of Medicine, Northwestern University, Chicago, IL, United States; ^5^School of Allied Health, College of Science, Health and Engineering, La Trobe University, Melbourne, VIC, Australia

**Keywords:** developmental origins of health and disease, early-life stress, child trauma, adverse childhood experiences, brain programming, self-regulation, developmental cascades

## Abstract

Early-life stress (ELS) poses risks for developmental and mental health problems throughout the lifespan. More research is needed regarding how specific ELS experiences influence specific aspects of neurodevelopment. We examined the association between ELS, defined as severe adversity (e.g., domestic violence, caregiver drug use) and severe relational poverty (e.g., caregiver neglect, lack of caregiver attunement), occurring during the first 2 months of life and a variety of brain-related, clinician-rated functions, including self-regulation and relational capacities. Interdisciplinary clinicians using the Neurosequential Model of Therapeutics (NMT), an approach to clinical problem solving, reported on the timing and type of treatment-seeking children’s (*N* = 2,155; 8–10 years) stressful experiences during four developmental periods: Perinatal (0–2 months), Infancy (2–12 months), Early Childhood (13 months to 4 years), and Childhood (4–11 years). They also reported on children’s current functioning in 32 brain-related domains (e.g., sleep, arousal, impulsivity, empathy, concrete cognition). Non-negative matrix factorization (NMF) was conducted on the 32 brain-related domains to identify latent factors, yielding four factors comprising Sensory Integration, Self-Regulation, Relational, and Cognitive functioning. Regularized hierarchical models were then used to identify associations between ELS and each latent factor while controlling for stress occurring during subsequent developmental periods, and children’s current degree of relational health. ELS (stress occurring during the first 2 months of life), specifically a severe lack of positive relational experiences (e.g., caregiver neglect, lack of caregiver attunement), was associated with the Sensory Integration and Self-Regulation factors. The Relational factor was better explained by stress occurring during childhood, and the Cognitive factor by stress occurring during infancy and childhood. Implications for how the timing and type of stress experiences may influence brain-related outcomes that are observed in clinical settings are discussed. Future directions include longitudinal follow-ups and greater specification of environmental variables, such as types of interventions received and when they were received, that may interact with ELS experiences to influence brain-related outcomes.

## Introduction

Research in animal models over the last 40 years has demonstrated that stressors experienced early in life can alter key neural networks in, and functioning of, the developing brain (Bath et al., 2013; Bedrosian et al., 2018). Similarly, extremes of developmental experience in humans (e.g., maltreatment and “adverse childhood experiences” or ACEs) that would plausibly create extreme or chaotic patterns of stress activation in the young child (i.e., traumatic stress) have been correlated with a wide range of pathology throughout the lifespan (Anda et al., 2006). In the more recent past, child trauma has been deemed a “public health crisis” by researchers (Magruder et al., 2016) and major news outlets (Blakemore, 2018), while public systems in early childhood, education, mental health, child welfare, juvenile justice and health are pushing to define and create “trauma-informed” practice, programs and policy (Pachter et al., 2017). Despite these intensive and expensive efforts in policy and practice, relatively little is known about the specific mechanisms by which early-life stress (ELS) or more broadly “ACEs” influence development to create the cascade of correlated functional consequences (Maniam et al., 2014).

Research from animal (Bedrosian et al., 2018) and human (Essex et al., 2013; Hambrick et al., 2018) models suggest that ELS, or stress occurring during the first days, weeks and months of life, may be particularly influential for neurodevelopmental or brain-related outcomes. This is because the stress occurs at a time when brain systems are rapidly organizing (i.e., during sensitive periods) and thus, at a time when the genotype is interacting with the environment to begin determining the most adaptive phenotypic expression (Hunter, 2012). Yet, the multi- and equifinality of problems that can occur following exposure to ELS make understanding exactly how ELS influences outcomes challenging, and may be part of the reason why children with early and pervasive stress experiences often exhibit lifespan impairment even when they receive intervention (Cakir et al., 2016; Williams et al., 2016). One-size-fits-all interventions do not reliably work for this subset of children who present with a diverse set of problems that can include severe sensory sensitivities, impulsivity and regulatory problems, relational impairments, and cognitive deficits (Perry, 2017).

One reason for the diversity of outcomes following stress exposure is that although each month of a child’s life comprises an equal amount of time, the influence that an experience can have on development in a given month changes as a child ages. Dynamic systems theory suggests that the more rapidly moving a dynamic system is, the more influence a perturbation will have (Thelen and Smith, 1998). Not all intervals of time have equal valence when it comes to the impact of experience. Tiny changes in a fundamental process can have a potentially lifelong echo because these decision points in differentiation essentially choose the direction for the subsequent organization of a system. From a clinical perspective, children with ELS are challenging to treat because we often notice their impairments long after the impaired systems have moved past a time of dynamic change. And, even tiny variations in the timing, pattern, and nature of the stress experiences may result in profound differences in how the effects of stress are manifest in clinical settings.

Moreover, although we have learned that ELS can alter developmental trajectories, “ELS” is itself a diverse concept. In applied research, ELS is often operationalized as children younger than 3–5 years (e.g., Dunn et al., 2017). Yet from birth to five, nearly 90% of brain development occurs, much neuroarchitectural structure has been built (Knudsen, 2004), and much phenotypic canalization has occurred. In addition, the very nature of a dynamic system is one that develops in response to its environment (Tronick and Hunter, 2016), making it key to specify both stress experiences and the context in which they occurred when evaluating how ELS influences outcomes. For example, a lack of relational buffers to stress experiences during the first weeks and months of life may be more likely to significantly sensitize the stress response system than a lack of relational buffers during any other period (DiCorcia and Tronick, 2011).

In addition, the outcomes evaluated when examining how ELS influences development have been diverse, ranging from HPA axis reactivity (Vargas et al., 2016) to risk for DSM-specified diagnoses (Saleh et al., 2017). In clinical populations, two improvements in evaluating how ELS influences outcomes are needed: evaluating how stress within more precise windows of development influences outcomes, and evaluating outcomes that are broadly brain-related and not restricted to DSM nosology. When we look with broad strokes, we may miss how specific insults occurring during specific periods of time influence specific outcomes (Andersen, 2003; Jensen et al., 2018). In addition, when we restrict outcomes to DSM diagnostic categories, we may miss the ways that ELS can confer risk for a variety of clinically relevant problems.

In this study, we utilize a dataset collected for clinical purposes that provides the advantage of containing detailed information regarding children’s developmental histories; specifically, the timing of both their adverse experiences and relationally positive experiences. Thus, the dataset allows for a fine-grained analysis of how ELS influences outcomes when controlling or accounting for stress occurring during later developmental periods. The dataset also contains clinician-ratings of items pertaining to children’s current functioning in 32 diverse brain-related domains (e.g., sleep, arousal, motor control, impulsivity, concrete cognition) that provide the ability to begin parsing out which clusters of brain-related functions ELS specifically influences.

## The Neurosequential Model of Therapeutics (NMT)

Data for this study were obtained from clinical metrics completed by clinicians utilizing the Neurosequential Model of Therapeutics (NMT). To contextualize the data, a brief description of the NMT follows. The NMT is an approach to clinical problem solving that allows clinicians to catalog the child’s developmental history and current functioning using the NMT Metrics. The output that clinicians receive following completion of the Metrics provides them with information for intervention planning including: (1) an estimate of the child’s developmental risk (a composite of adverse experiences and relational poverty) throughout several developmental periods; (2) the child’s degree of current relational health; and (3) information regarding the child’s current brain-related functioning parsed out into a total “central nervous system (CNS) Functioning” score and four subdomains: Sensory Integration, Self-Regulation, Relational, and Cognitive. Moreover, the functional capacities that clinicians report on are clustered from “lowest” in the brain and “first” to organize (sensory integration) to the “top” of the brain and “last” to fully organize (cognitive). This organization is based on the primary heuristic of the Neurosequential Model, a sequentially-organizing functional model of the brain (Perry, 2006).

Indeed, we have found this clinical heuristic to be helpful to providers because it creates a mechanism by which the Metric output can be organized. The four subdomains were selected to broadly yet efficiently represent primary aspects of brain-related functioning that are mediated by “lower” (e.g., autonomic regulation, metabolism, and other functions that may facilitate sensory processes), as opposed to “higher” (e.g., abstract/reflective cognition, math/symbolic cognition, and other functions that may facilitate cognitive processes) parts of the brain. Central to the NMT is the notion that clinical problems are brain-related; that a better understanding of neurodevelopment can facilitate clinical insight. Also central is the appreciation that the brain develops, in part, in a use-dependent fashion, making careful ascertainment of developmental histories paramount in the clinical decision-making process.

The NMT has been named an “emerging practice” by the National Quality Improvement Center for Adoption/Guardianship Support and Preservation (QIC-ag.org/). More information on the NMT/NMT Metrics is detailed elsewhere (Perry and Hambrick, 2008; Perry, 2014).

## Current Study

In this study, we used a large clinic-referred sample to begin to examine how ELS, which we define as stress occurring during the first 2 months of life, influences the brain-related outcomes captured in the NMT compared to stress occurring later in infancy and childhood. We simultaneously accounted for and examined the effects of severe stress occurring during subsequent developmental periods on the same functions. Although various conceptualizations of ELS have included the period up to two, three, and even 5 years of age (e.g., Ogle et al., 2013; Dunn et al., 2017), we operationally defined ELS in this study as the first 2 months of life given research suggesting that the first few months of life may be the most rapid time of *ex-utero* brain growth and thus a sensitive period for a variety of experience-dependent outcomes (e.g., Kuzawa et al., 2014).

Very early life experiences can broadly affect development and are often cited as a risk factor for a variety of chronic and severe health and behavioral health problems throughout the lifespan (Maniam et al., 2014). However, in clinical samples, we need to better understand which types of ELS confer risk and for which outcomes while accounting for stressors experienced later in development. We hypothesized that consistent with other findings using this dataset (Hambrick et al., 2018, 2019), ELS would have a strong association with most brain-related functions given the oftentimes pervasiveness of the effects of early life experience on brain-related functions. However, we also hypothesized that early life experiences would have some specific effects on certain brain-related functions. We expected both types of ELS, severe adversities and severe relational poverty, to be associated with self-regulatory functions, which may develop very early in life (Stiles and Jernigan, 2010) and be highly sensitive to stressors and a lack of co-regulatory experiences (DiCorcia and Tronick, 2011; Beeghly et al., 2016). We did not expect ELS experiences to have as strong of an influence on brain-related functions that were more cognitive in nature, which may have periods of dynamic growth that are longer and perhaps most dynamic later in life (Andersen, 2003).

## Materials and Methods

### Design

Data were obtained from the NMT Clinical Practice Tools (henceforth NMT Metrics). NMT Metrics are completed by clinicians using the NMT as part of their clinical practice (Perry, 2009). De-identified NMT Metric data were downloaded from the web-based repository of data housed by the Neurosequential Network (NMT developers) for quality improvement purposes. This study was deemed “Not Human Subjects Research” by the lead author’s institutional review board.

To complete the Metrics, clinicians must report on the timing, severity, and type of a child’s stress experiences across several developmental periods, from the intrauterine period through the current age of the index client being assessed. The developmental periods relevant to this study are: Perinatal (birth to 2 months), Infancy (2–12 months), Early Childhood (13 months to 4 years), and Childhood (4–11 years). Clinicians then report on the quality of a child’s relationally positive experiences in like manner. These developmental periods are not exhaustive, but were selected by NMT developers to balance two objectives: (1) ease of clinician Metric use; and (2) age groups that allow for the most specificity during very early development (i.e., the first 3 years of life), when child development is nearly logarithmic (Johnson, 2001).

Next, clinicians report on a child’s current functioning in 32 brain-related capacities (e.g., attention, impulse-control, affect regulation, fine motor control). These 32 capacities are represented both as a total “CNS Total” score and are then clustered into four broader “domain” scores (sensory integration, self-regulation, relational, cognitive) based upon the sequential organization and development of the brain.

Clinicians are provided with extensive training in Metric use throughout the certification process (Phase I certification is approximately 150 h). NMT Trainers from the Neurosequential Network conduct biannual Fidelity Exercises, where all Metric users are given the same case (client) data with which to complete the metrics. Clinician performance in the Fidelity Exercise yields a fidelity rating of None, Low, Acceptable, or High. This rating reflects the degree of interrater reliability between the clinician and NMT developers. Clinicians whose Metrics were included in this study were NMT Phase I Certified or in advanced stages of completing the certification process, and had achieved an “acceptable” or “high” fidelity rating.

### Participants

NMT Metric data from 2,155 children ages 8–10 years (*M* = 9.40, SD = 0.89) seeking behavioral health services with histories of developmental adversity were used. This age range was selected for two reasons; the first is that initial clinical presentation to the mental health system often occurs during this age range and, second, the sample number in this age range provided adequate numbers to support the factor analysis used. Table 1 contains additional sample descriptives. Data were collected from clinicians across 190 diverse clinical “sites” across the US, Canada, Europe, and Australia. Because both sites and individuals can be NMT certified, most “sites” were a single clinician, while other sites contained ratings from multiple clinicians. Most sites are primarily outpatient, while some are a mixture of outpatient and residential/inpatient. One site was comprised of metrics completed by the NMT developers. At all sites, a percentage of patients were child welfare-involved, ranging from 10% to 100%.

**Table 1:** Descriptives.

	
Female (%)	33.27
White (%)	61.68
Asian (%)	1.53
Black (%)	16.75
Hispanic (%)	6.82
Native American (%)	1.76
Other (%)	16.56
*N*	2,155

Most clinician characteristics are unknown. However, all NMT-certificated clinicians have a master’s degree in a relevant clinical discipline (e.g., nursing, social work, psychology) and hold an active license. Approximately 20% of NMT clinicians have more advanced degrees (e.g., PhD, PsyD, DNP, MD). The NMT certification process takes approximately 150 h and includes specific training to calibrate providers’ ratings of the Metric items.

### Measures

#### NMT Metrics

The NMT Metrics are divided into four parts: Part A (severity of “nodal” traumas, adversities and stress experiences across several developmental periods), Part B (quality of relational experiences across several developmental periods), Part C (current brain-related functioning, comprising items developed to measure 32 different capabilities which are subsequently clustered into four domains: sensory integration, self-regulation, relational, and cognitive functioning), and Part D (current relational health). Although the Metrics are completed by clinicians, clinicians are instructed to use information from clinical interviews, child welfare case files, observations of child/family, medical records, psychosocial assessments, and any other reliable source of information while completing them.

In Part A (stress and adversity), clinicians report whether a child experienced a range of potentially traumatic, adverse or stressful experiences during the following periods: Perinatal (0–2 months), Infancy (2–12 months), Early Childhood (13 months to 4 years), and Childhood (4–11 years). The six experiences assessed per developmental period are quality of primary caregiving, caregiver drug/alcohol use, neglect, domestic violence, transitions/chaos and “other trauma” (e.g., natural disaster, gun violence). Clinicians rate the severity of each experience from 1 to 12, ranging from None/Minimal (1–3), Mild (4–6), Moderate (7–9), to Severe (10–12). When clinicians are uncertain about the severity of a child’s experience, they are instructed to provide a “neutral” score (6 or 7), to use clinical reconstruction to estimate if the score should be marked up (more severe) or down (less severe) by a few points given what is known about the overall nature of the child’s early experiences, and to ultimately underestimate the potential risk. Given these scoring instructions, scores falling in the range of 10–12 are highly likely to reflect documented, profoundly severe traumas, stressors or adversities.

In Part B (relational experiences), clinicians report on the quality of a child’s relationships across the same developmental periods. The six experiences assessed per period are primary caregiver safety, primary caregiver attunement, consistency in primary caregiving, paternal (or partner) support, kinship support, and community support on a scale of 1–12 from Poor (1–3), Episodic (4–6), Adequate (7–9), to Positive (10–12). These items were created to assess quality of caregiving and overall “social support” but also, particularly in early developmental periods, risk for attachment disruption. The same scoring instructions are used to complete Parts A and Part B. Therefore, Part B scores ranging from 1 to 3 are likely to indicate profound absence of co-regulatory experiences and relational health. Although some items in Part A and Part B are similar, clinicians use a different lens when completing each section. In Part A, they report adversities, where in Part B, they report the strength of a child’s relational health.

Part C (current CNS Functioning) is clinician rating of a child’s capabilities across brain-related functions. Functions span from basic autonomic regulation, such as cardiovascular regulation to sleep, feeding/appetite, fine motor skills, affect regulation, relational skills, arousal, ability to modulate reactivity/inhibit impulsivity, and abstract/reflective thinking skills. When completing their ratings, clinicians are asked to review (when possible) medical records, and also gather history from caregivers about medical conditions. In addition, many NMT-certified clinicians—particularly nurses and other medical professionals—obtain heart rate and blood pressure data as part of their clinic visits. There are also specific scoring “rules” that clinicians learn, such as to assume “typical” cardiovascular regulation unless they obtain history or data suggesting otherwise. Clinicians rate whether a child’s capabilities are “age typical” or whether they fall above or below age typical on the 32 items comprising the Part C checklist on a scale of 1–12, from Severe Dysfunction (1–3), Moderate Dysfunction (4–6), Mild Dysfunction (7–9), and Normal Range (10–12). The highest CNS Functioning score is 384, and represents the capacity of a “typical” adult. This score should not be interpreted like an IQ score. A score of 384 indicates a general lack of dysfunction in the measured brain-related capacities, not “above average” nor “exceptional” functioning.

Part D (current relational health) is clinician rating of the quality of a child’s current relational context across nine domains, including primary caregivers, siblings, extended family, school, peers, and community. Clinicians rate the quality of each of the child’s current relational experiences from Poor (1–3), Episodic (4–6), Adequate (7–9) to Positive (10–12). Then, these nine items are summed to create a total Current relational health score.

Regarding the reliability and validity of the NMT metrics, in a sample of children with fetal alcohol spectrum disorders, improvements in total CNS Functioning following 6 months of NMT-guided intervention were associated with improvements in scores on the Battelle Developmental Inventory–2nd Ed (BDI-2) and the Parenting Stress Inventory (PSI; Zarnegar et al., 2016); *r* = 0.67 between the BDI-2 total score and CNS Functioning; *r* = −0.38 between the PSI total score and CNS Functioning. Significant correlations between Part C items and the Trauma Symptom Checklist for Young Children Posttraumatic Stress Total score include arousal (*r* = −0.408) and modulate reactivity/inhibit impulsivity (*r* = −0.390; Jackson et al., 2016). In an analysis using a subsample of the current dataset, Cronbach’s α was 0.95 for Part C, and was 0.85 for Part D (Hambrick et al., 2018). In a study using qSPECT to examine brain regional perfusion and individual brain-related functional items in the NMT Brain Map (18 children and youth with histories of maltreatment; ages 5–18), there was significant correlation between the brain regions demonstrating abnormal perfusion (Z scores >2 or <−2) and “atypical” functional scores in specific items associated with comparable brain areas in the NMT Brain Map heuristic. These findings support the validity of the NMT Metric constructs (Quint et al., 2019). In addition, in statistical models, site bias of CNS Functioning ratings has been shown to be statistically indistinguishable from the ratings of the NMT developers (Hambrick et al., 2018).

In this study, Cronbach’s α was 0.95 for Part C (current CNS functioning) 0.85 for Part D (current relational health). Cronbach’s α was not computed for Parts A (stress/adversity) nor B (relational experiences), because this is an inappropriate statistic when an endorsement of one item does not necessarily increase the likelihood of endorsement on other items (Bollen and Bauldry, 2011).

### Data Analysis

#### Operationalization of Variables

Using our sample of 8- to 10-year-olds, we first created numerical predictors to represent the stress experiences across Parts A and B. Clinicians are instructed to only use scores ranging from 10 to 12 (Part A) and 1–3 (Part B) when the experience was truly profound and can be corroborated. Thus, we created count indicators of the number of experiences rated 10–12 in Part A (severe adversity/stress), and also 1–3 in Part B (poverty of relational experiences), per child, per developmental period. The independent variables (IVs) for Perinatal—Adversity/Stress ranged from 0 to 6 (six possible stressors are assessed per developmental period). The IVs for Perinatal—Relational Experiences also ranged from 0 to 6. Part D scores were summed to create a broad indicator of a child’s current degree of relational health to be used as a covariate. Variable names and how they were used in the regression analysis are listed in Table 2.

**Table 2:** Variables in regression analysis.

Type	Variable name	Variable explanation
Control	Age in Months	Child age in months
	Gender	Male (reference), Female
	Race/Ethnicity	White (reference), Asian,
		Black, Hispanic, Native
		American, Other
	Site	Binary indicator of clinical site
Independent	Intrauterine	Degree of intrauterine
	Drug/Alcohol	drug/alcohol exposure: 1
		(none), 12 (severe)
	Current Relational	Degree of current relational
	Health	health: 1 (poor), 12 (positive)
	Perinatal—Adversity/Stress	Number of experiences rated
		10–12 in Perinatal Part A: 0–6
	Perinatal—Relational	Number of experiences rated
	Experiences	10–12 in Perinatal Part B: 0–6
	Infancy—Adversity/Stress	“”
	Infancy—Relational	“”
	Experiences
	Early	“”
	Childhood—Adversity/Stress
	Early	“”
	Childhood—Relational
	Experiences
	Childhood—Adversity/Stress	“”
	Childhood—Relational	“”
	Experiences
	Youth—Adversity/Stress	“”
	Youth—Relational	“”
	Experiences
Dependent	Factor 1	Self-Regulation
	Factor 2	Sensory Integration
	Factor 3	Cognitive
	Factor 4	Relational

#### Descriptive Analyses

We examined frequencies of and Spearman correlations between indicators of severe adversity/stress (Part A) and poverty of relational experiences (Part B) throughout the developmental periods.

#### Factor Analysis

We conducted non-negative matrix factorization (NMF) to identify the most salient latent factors within the 32 measured brain-related (Part C) functions. Although we could have used the exact factors (Self-Regulation, etc.) that drive the NMT clinical decision-making process, we chose to conduct a statistically-driven factor analysis to promote our ability to identify the most salient subcategories of functioning captured by the 32 items in Part C. Matrix decomposition *via* NMF, as contrasted to principal components analysis, requires strictly positive input and learns strictly positive latent factors, implying that the matrix reconstruction occurs only through an additive linear combination of the factors (Lee and Seung, 1999). This decomposition method is beneficial in that the factors themselves have a lower bound at 0 and thus intuitively represent the degree to which each of the items in the scale and the observations in the data are present across the latent variables. Because the measured brain-related functions meet the non-negativity constraint, we found that the non-negative factors obtained by NMF facilitated interpretation of the latent categories of interest.

#### Correlation and Regression Analyses

Next, we examined frequencies of and Pearson correlations between the latent factors. In the regression analyses, we controlled for severity of intrauterine substance abuse (scale of 1–12; 12 = severe intrauterine use/abuse). When completing Part A, clinicians also rate intrauterine experiences. Given the low degree of clinician-rated “confidence” in the other assessed intrauterine experiences, this was the only item included from the intrauterine scale. Other controls included demographic attributes: age (months), and binary indicators of gender (female = 1), race/ethnicity, and each site.

The four regression analyses are regularized hierarchical linear models of each latent factor as a function of the stress/adversity (Part A) and relational experiences (Part B) scores for each of the developmental periods, as well as the control variables (including current relational health and the other latent factors). Importantly, the correlation among the adversity/stress and relational experiences scores across developmental periods produced concern for multicollinearity. We found further evidence of multicollinearity in the condition numbers, which hovered around 24.4, and are therefore indicative of unstable regression coefficients (Fox, 2008). To address this, we used ridge regularization, which helps reduce the variance of estimates due to multicollinearity (Hastie et al., 2009). Because we chose the degree of penalization through cross-validation, regularization also helps prevent overfitting (Type I Errors).

Binary indicators of site address the potential nesting of observations within sites and produce a hierarchical linear model. With no regularization, such a model often inflates the estimated differences among sites and produces Type 1 Errors specific to the site estimates (Gelman and Hill, 2007). Gelman and Hill instead suggest partial pooling of the intercepts, where site effects are assumed to follow a Normal distribution centered at 0. In our case, we fit linear regression models with regularizing Gaussian priors on the site intercepts, in addition to all other parameters in the model, producing a hierarchical linear model with partial pooling of site effects.

Two consequences of our regularization strategy are relevant for interpreting the regression estimates. First, analytical standard errors are not available, and therefore we evaluate uncertainty in the coefficient estimates using 95% accelerated bootstrap confidence intervals (Efron, 1987). Second, to penalize the regression estimates in the model equally, all IVs are standardized to the same scale. Therefore, unit changes in the standardized IVs correspond to one standard deviation, and estimates should be interpreted as the expected change in the latent factor due to a change of one standard deviation in the IV in question.

## Results

### Descriptive and Correlation Analyses

**Figure 1:**
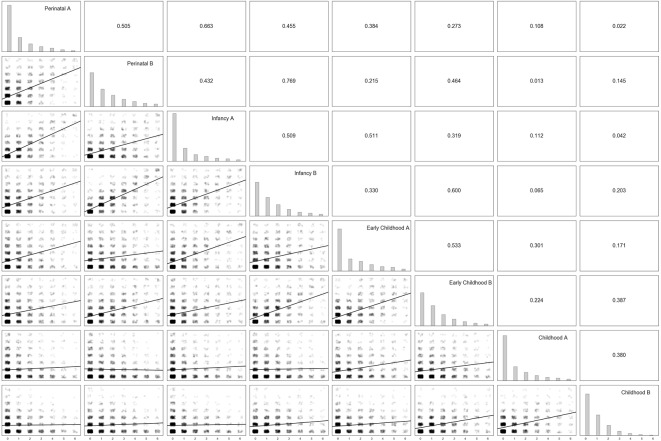
Parts A and B correlation matrix. Note. On the diagonal from top left to bottom right, frequencies of severe Part A (adversity/stress) experiences and Part B (poor relational) experiences per developmental period. In the lower triangle, jittered bivariate scatter plots for each combination of Part A and B stress severity indicators, along with the line of best fit. In the upper triangle, the corresponding Spearman correlation coefficients for each combination of Part A and B stress severity indicators.

The degree of severe adversity/stress and relational poverty experiences was similar across all developmental periods and across Parts A and B (Figure 1). Correlations between stress indicators across developmental periods were typically strongest for more proximal developmental periods (Figure 1; correlations ranged from 0.752 (Perinatal Adversity/Stress and Perinatal Relational Experiences) and 0.022 (Perinatal Adversity/Stress and Childhood Relational Experiences).

### Factor Analysis

We specified four latent factors in the NMF model for theoretical and statistical reasons. Theoretically, Part C was developed to assess four domains of brain-related functioning: sensory integration, self-regulation, relational, and cognitive. Statistically, when we tested models specifying more than four factors, additional factors beyond the four presented here were less clearly defined in theoretical terms, and the factor loadings for the additional factors were substantially weaker.

**Figure 2:**
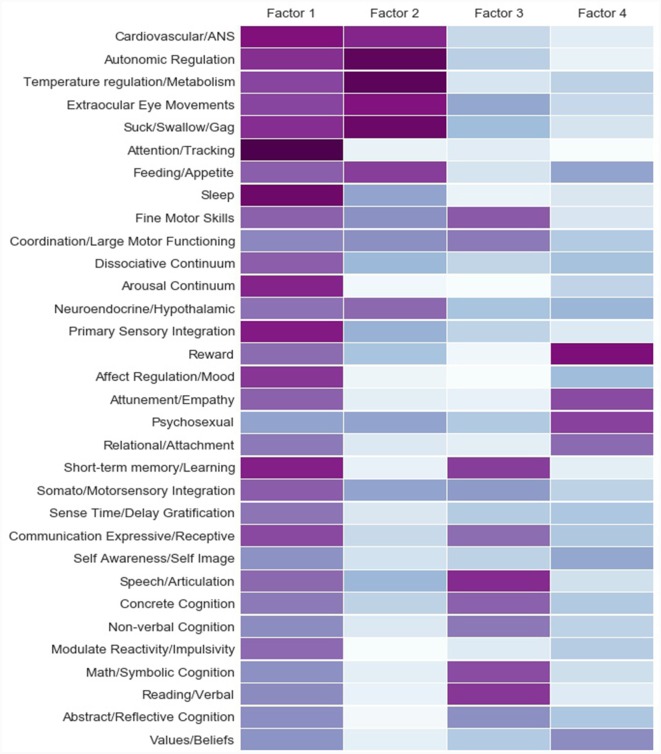
Factorization. Note. Factor loadings from the Non-negative Matrix Factorization (NMF) of the Part C (brain-related) items of the Neurosequential Model of Therapeutics (NMT) Metrics. Metrics were obtained from 8- to 10-year-old treatment-seeking children and represented the child’s current functioning across a variety of brain-related functions. Individual Part C items are represented on the left. Light blue represents weak and dark purple strong factor loadings. Factor 1 = Self-Regulation, Factor 2 = Sensory Integration, Factor 3 = Cognitive, Factor 4 = Relational.

Factor 1 (all factors depicted in Figure 2) can be characterized as an indicator of self-regulatory functions (e.g., Sleep, Arousal, Attention/Tracking, Primary Sensory Integration, Affect Regulation/Mood). These functions tend to involve a set of primary regulatory networks (Tronick and Perry, 2015). This factor accounted for more variance and contained more substantial factor loadings than any other factor.

Factor 2 appeared comprised of functions underlying primary sensory integration capacities, many of which are brainstem-related and likely have significant *in utero* and very early life organization (e.g., Autonomic Regulation, Temperature Regulation/Metabolism, Suck/Swallow/Gag).

Factor 3 appeared comprised of mostly cognitive functions given that items developed to measure concrete and abstract cognitive functions (e.g., Speech/Articulation, Concrete Cognition, Math/Symbolic Cognition, Reading/Verbal) loaded strongly on this factor; also noteworthy were strong loadings of Fine Motor Skills and Coordination/Large Motor Functioning on this factor, given that the cerebellum has been increasingly recognized for its role in cognition, including language (Buckner, 2013).

Factor 4 appeared mostly comprised of items intended to measure relational functions (Reward, Attunement/Empathy, Psychosexual, Relational/Attachment). Interestingly, the Values/Beliefs item, intended to measure higher-order cognitive skills, loaded most strongly on this factor, potentially indicating significant relational mediation of this cognitive skill. This was the factor that explained the least variance in the data.

**Figure 3:**
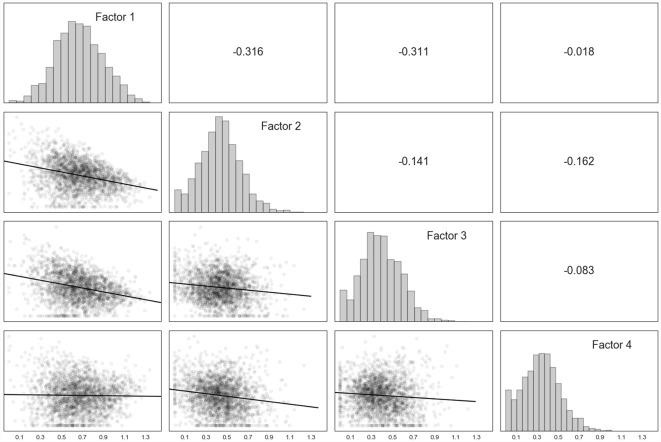
Factor correlation matrix. Note. On the diagonal from top left to bottom right, frequencies for each of the four factors derived from Part C (brain-related) items identified by the non-negative matrix factorization. In the lower triangle, bivariate scatter plots for each combination of the four factors, along with the line of best fit. In the upper triangle, Pearson correlation coefficients for each combination of the factors. Factor 1 = Self-Regulation, Factor 2 = Sensory Integration, Factor 3 = Cognitive, Factor 4 = Relational.

The factors appeared relatively normally distributed (Figure 3). Factor 1 was moderately correlated with Factor 2, and Factor 2 with Factor 3. Due to most factors being comprised of the items that they were developed to be comprised of (Perry, 2006; Perry and Dobson, 2013), we henceforth use the descriptors of Self-Regulation (Factor 1), Sensory Integration (Factor 2), Cognitive (Factor 3), and Relational (Factor 4).

### Regression Analyses

Consistent with hypotheses, regression analyses (Tables s 3–6) demonstrated a significant negative influence of ELS (stress occurring during the first 2 months of life or the “perinatal” period), specifically negative Perinatal Relational Experiences, on two of the latent factors: Self-Regulation (Factor 1, Table 3) and Sensory Integration (Factor 2, Table 4). Also consistent with hypotheses, ELS was not related to the Cognitive Factor (Factor 3, Table 5). Perhaps surprisingly, ELS was also not related to the Relational factor (Factor 4, Table 6).

**Table 3:** Hierarchical ridge regression: early life stress (ELS) and self-regulation outcomes, Factor 1.

	Estimate	CI lower	CI upper
Intrauterine Drug/Alcohol	−0.0044	−0.0124	0.0041
**Current Relational Health**	**0.0735**	**0.0626**	**0.0837**
Perinatal—Adversity/Stress	−0.0091	−0.0235	0.0032
**Perinatal—Relational Experiences**	**−0.0191**	**−0.0321**	**−0.0063**
**Infancy—Adversity/Stress**	**−0.0168**	**−0.0308**	**−0.0019**
Infancy—Relational Experiences	0.0005	−0.0133	0.014
**Early Childhood—Adversity/Stress**	**−0.0167**	**−0.0273**	**−0.0057**
Early Childhood—Relational Experiences	0.0027	−0.0091	0.015
Childhood—Adversity/Stress	0.0048	−0.0045	0.014
Childhood—Relational Experiences	0.0012	−0.0084	0.0109
**Age in Months**	**0.0095**	**0.0025**	**0.0169**
**Female**	**0.0197**	**0.0125**	**0.0265**
Asian	0.0015	−0.0073	0.0104
Black	−0.0041	−0.0127	0.0035
Hispanic	−0.0007	−0.0085	0.008
Native American	−0.0044	−0.0137	0.0046
Other	−0.0038	−0.012	0.0045
**Factor 2 (“Sensory Integration”)**	**−0.45**	**−0.4944**	**−0.4044**
**Factor 3 (“Cognitive”)**	**−0.3794**	**−0.4201**	**−0.3363**
**Factor 4 (“Relational”)**	**−0.303**	**−0.3567**	**−0.2552**

*Note. Adversity/Stress and Relational Experiences scores are ordinal indicators of the number of adversity or relational health indicators within the “severe” or “poor,” respectively, ranges. Site indicators are not represented here for brevity (190 site indicators). For ethnic/racial indicators, White is the reference category. Bolded findings are significant*.

**Table 4:** Hierarchical ridge regression: ELS and sensory integration, Factor 2.

	Estimate	CI lower	CI upper
Intrauterine Drug/Alcohol	−0.005	−0.0131	0.0037
**Current Relational Health**	**0.0216**	**0.0121**	**0.0313**
Perinatal—Adversity/Stress	−0.0076	−0.0203	0.0049
**Perinatal—Relational Experiences**	**−0.0155**	**−0.0285**	**−0.0036**
Infancy—Adversity/Stress	−0.0095	−0.0219	0.0025
Infancy—Relational Experiences	0.0074	−0.0065	0.0202
**Early Childhood—Adversity/Stress**	**−0.0108**	**−0.0207**	**−0.0006**
Early Childhood—Relational Experiences	0.0035	−0.0078	0.015
Childhood—Adversity/Stress	0.0011	−0.0073	0.0098
Childhood—Relational Experiences	0.0051	−0.0033	0.0135
Age in Months	0.0029	−0.0042	0.0105
Female	−0.0061	−0.0136	0.0013
Asian	−0.0026	−0.0097	0.0043
**Black**	**0.0111**	**0.0037**	**0.0199**
Hispanic	−0.0012	−0.0087	0.0063
Native American	−0.0034	−0.0106	0.0049
Other	−0.0006	−0.0088	0.0073
**Factor 1 (“Self-Regulation”)**	−0.4237	−0.4648	−0.3811
**Factor 3 (“Cognitive”)**	−0.3032	−0.3504	−0.2594
**Factor 4 (“Relational”)**	**−0.2617**	**−0.3107**	**−0.2139**

**Table 5:** Hierarchical ridge regression: ELS and cognitive outcomes, Factor 3.

	Estimate	CI lower	CI upper
Intrauterine Drug/Alcohol	−0.0077	−0.0166	0.0011
Current Relational Health	0.0069	−0.0034	0.0174
Perinatal—Adversity/Stress	−0.0056	−0.0201	0.0085
Perinatal—Relational Experiences	−0.0058	−0.0203	0.0084
**Infancy—Adversity/Stress**	**−0.0172**	**−0.0324**	**−0.0032**
Infancy—Relational Experiences	0.0055	−0.009	0.0193
Early Childhood—Adversity/Stress	0.0005	−0.0103	0.0109
Early Childhood—Relational Experiences	−0.0039	−0.0161	0.0075
**Childhood—Adversity/Stress**	**0.0141**	**0.0052**	**0.0221**
Childhood—Relational Experiences	−0.0102	−0.0193	0
Age in Months	0.0069	0	0.014
Female	0.007	−0.0013	0.0146
Asian	0.0023	−0.005	0.0091
Black	0.0035	−0.005	0.0118
Hispanic	−0.0068	−0.0155	0.0016
Native American	−0.0021	−0.012	0.0075
Other	0.0039	−0.0041	0.0127
**Factor 1 (“Self-Regulation”)**	**−0.3852**	**−0.4337**	**−0.3398**
**Factor 2 (“Sensory Integration”)**	**−0.3266**	**−0.3763**	**−0.2777**
**Factor 4 (“Relational”)**	**−0.1832**	**−0.2395**	**−0.1292**

**Table 6:** Hierarchical ridge regression: ELS and relational outcomes, Factor 4.

	Estimate	CI lower	CI upper
Intrauterine Drug/Alcohol	0.0017	−0.0065	0.0093
**Current Relational Health**	**0.0753**	**0.0654**	**0.0842**
Perinatal—Adversity/Stress	−0.0115	−0.0238	0.001
Perinatal—Relational Experiences	−0.0024	−0.0152	0.0094
Infancy—Adversity/Stress	−0.0066	−0.0185	0.0053
Infancy—Relational Experiences	−0.0024	−0.0149	0.01
Early Childhood—Adversity/Stress	−0.0108	−0.0213	0.0002
Early Childhood—Relational Experiences	−0.0037	−0.0148	0.0075
**Childhood—Adversity/Stress**	**−0.0094**	**−0.0181**	**−0.0017**
Childhood—Relational Experiences	0.0008	−0.0083	0.0098
Age in Months	0.0004	−0.0059	0.0073
**Female**	**−0.0136**	**−0.0206**	**−0.0061**
Asian	0.0068	0.0002	0.0133
Black	0.0121	0.0049	0.0198
Hispanic	0.01	0.0022	0.0173
Native American	−0.0015	−0.0107	0.0081
Other	0.006	−0.0013	0.0141
**Factor 1 (“Self-Regulation”)**	**−0.2549**	**−0.2986**	**−0.2115**
**Factor 2 (“Sensory Integration”)**	**−0.2332**	**−0.2792**	**−0.1901**
**Factor 3 (“Cognitive”)**	**−0.1528**	**−0.1974**	**−0.108**

There were other noteworthy findings beyond those specific to ELS (stress occurring during the Perinatal period). Some stress experiences during later developmental periods (Infancy, Early Childhood, Childhood) were also associated with outcomes. Regarding Self-Regulation, Adversity/Stress during Infancy and Early Childhood predicted poorer functioning (Table 3), whereas having a high degree of current relational health predicted better functioning. Regarding Sensory Integration (Table 4), Adversity/Stress during Early Childhood predicted poorer functioning, while Current Relational Health was again protective. For the Cognitive factor (Table 5), Adversity/Stress during Infancy predicted poorer functioning, while perhaps surprisingly, Adversity/Stress during Childhood predicted better functioning. Current Relational Health was not protective for Cognitive outcomes. Regarding the Relational factor (Table 6), Adversity/Stress during Childhood predicted poorer functioning, while Current Relational Health was protective.

## Discussion

The default assumption of a dynamic system is to organize, and so it does. In humans, the dynamic system of the brain organizes most rapidly in the earliest days and week of life, and most adaptively in the face of co-regulation (DiCorcia and Tronick, 2011). Yet, not all neurodevelopmental systems engage in rapid organization at the same time, and at the same rate (Jensen et al., 2018). The preponderance of recent evidence suggesting that the effects of ELS are strong and enduring has been an important finding for fields focused on the developmental origins of disease. Now, it is important to better understand which brain-related capacities are most affected by which types of ELS.

This article represents an attempt to identify brain-related capacities that appear to comprise various latent factors of function amongst a treatment-seeking sample of youth. It also seeks to identify how the timing and type of severe stress experiences influence said factors. Subsequent efforts to refine this study will leverage a dataset that is growing rapidly in size and permitting more granular inferences.

In this study, severe stress experiences occurring within four developmental periods (perinatal, infancy, early childhood, and childhood) were conceptualized in two ways: (1) counts of severe occurrences of “nodal” traumas, adversities, or stressors; and (2) counts of severe relational poverty experiences (e.g., lack of caregiver attunement and family/community support).

### Descriptive and Correlation Analyses

Results indicated that severe adversity/stress and poor relational experiences occurred with similar frequency across all developmental periods (Figure 1). This resonates with research suggesting that contrary to conventional wisdom, the first year of life is a time of significant risk for victimization (Turner et al., 2006). It also suggests that current findings regarding the strength of the association between ELS and outcomes are not due to our sample evidencing a disproportionate number of severe stress experiences during the first 2 months of life.

In addition, severe adversity/stress and poor relational experiences were correlated within developmental periods (Figure 1). Meaning, children experiencing severe stressors within a given developmental period were also likely to lack quality relational experiences during that period. We also found that negative experiences during one developmental period tended to predict negative experiences during other developmental periods when the periods were temporally proximate. Given that this was a treatment-seeking sample with a high percentage of child welfare-involved children, in many cases, children with severe ELS may have received services that led to their later developmental experiences becoming more positive, and *vice versa*. Unfortunately, the current dataset does not contain variables regarding, for example, dates of child welfare involvement nor dates/types of intervention services.

### Factor Analysis

After reviewing descriptive data, we conducted a factorization of the 32 brain-related functions reported on in the NMT Metrics. Although it is uncommon to refer to clinician-rated functions as “brain-related functions,” a goal of the NMT is to bring providers into keener awareness of how biological processes interact with developmental experience in ways that have significant intervention implications (Perry and Hambrick, 2008). Thus, because these functions are indeed brain-related, we use the NMT terminology here.

Results from the factorization suggested that, consistent with how the NMT Metrics were developed (Perry, 2006; Perry and Dobson, 2013), four interpretable latent factors emerged that could be roughly characterized as comprising Self-Regulation (Factor 1), Sensory Integration (Factor 2), Cognitive (Factor 3), and Relational (Factor 4) subdomains of brain-related function (Figure 2). Results of the factor analysis, although overall consistent with our hypotheses about which domains would emerge, were not totally consistent with how the NMT Metrics were developed. For example, motor skills loaded most strongly on the Cognitive factor, whereas it was originally hypothesized that they might load more strongly on the Sensory Integration factor. Values/Beliefs, an item developed to be cognitive in nature, loaded more strongly on the Relational factor. Thus, we view the current analysis as an important step in our conceptualization of how these brain-related functions cluster together and relate to the clinical presentations of children and youth.

None of the factors were strongly correlated (*r*’s were <0.315), suggestive of their distinctness (Figure 3). The Self-Regulation factor comprised the most variance and contained the strongest item loadings (Figure 2). This may be because individuals with strong self-regulatory capacities are likely to remain in a functional state that permits engagement in higher-order tasks, including tasks that are relationally and cognitively mediated (Denham et al., 2012). In addition, it was a bit of a surprise that the items measuring motor functioning loaded most strongly on the Cognitive factor. However, there is increasing consensus of the role of the cerebellum in higher-order cognition (Buckner, 2013). Finally, although it was not an express purpose of this article, this factorization lends some data-driven validity to the theoretical concepts underlying the NMT assessment process.

### Regression Analyses

Perhaps the most striking finding across the regressions was the fact that ELS was most detrimental to outcomes, specifically self-regulation (Table 3) and sensory integration (Table 4) when operationalized as negative relational experiences. This finding is unsurprising, however, given that children’s abilities to navigate their sensory environments and self-soothe begin to develop very early in life and through the context of attentive, sensitive co-regulatory caregiving experiences (Beeghly et al., 2016). This does not mean that “nodal” adversities or stressors do not matter during this developmental period, but that they do not matter as much as the quality of the relational experiences the infant receives.

It was interesting, in that regard, that ELS was not associated with relational functioning. Many have hypothesized that the roots of relational skills develop during the first year of life (Evans and Porter, 2009). While at the same time, children adopted prior to about 3 years of age or who receive remedial relational supports between 0 and 3 are often able to evidence fairly normative relational capacities even if their very early life experiences were suboptimal (Ghera et al., 2009). Thus, perhaps it is more detrimental to a child’s relational abilities if negative experiences persist throughout childhood. Indeed, we see some evidence of this in the current results; adversities/stressors during childhood were associated with poor relational outcomes, but not ELS. Another potential reason for this finding is that because the majority of children have some “unknowns” in their early life histories, we ask clinicians to “underestimate” the child’s potential risk. The number of cases where full confidence can be given to a serious relational problem is thus significantly diminished in this sample. In addition, the Relational factor evidenced the weakest factor loadings (Figure 3), making it potentially more challenging to predict relational functioning until the dataset grows. Regardless, current results suggest that there is some specificity in how the timing of stress influences specific brain-related outcomes.

Consistent with previous analyses using this dataset (Hambrick et al., 2018, 2019), a child’s degree of current relational health was a strong predictor of almost every aspect of neurodevelopmental function (except cognitive functioning). Readers are referred to the cited texts for more detailed descriptions of the potential meaning of this finding. In regard to the current analysis, it is an important finding that improving relationships later in life across family, school, peer and community domains may help buffer developmental risk.

Although a thorough review of how stressors experienced later in life may influence brain-related functioning is beyond the scope of this article, we note that, similar to findings from the Relational factor, stress during later developmental periods indeed exerts influence on functioning even when controlling for the influence of ELS; particularly stress occurring later in infancy and early childhood (Tables s 3–6). It will be important to think critically about how the timing of various stressors may interact with biological vulnerability to produce risk or protection for specific outcomes.

We also note the positive association between adversity/stress during childhood and cognitive outcomes. This is not as surprising as it may, at first, appear. One of the major adaptive responses in an inescapable, overwhelming experience is dissociation (Dutra et al., 2009). A young child in distress with an absent or unpredictable caregiver will tend to dissociate. If this becomes a preferred mechanism of coping, as the child grows, the “cognitive sparing” that occurs with dissociation will allow the child to have relatively more “typical” cognitive development and still have profound regulatory and relational problems. Indeed, relatively typical scores in the “cognitive” domain relative to the others are one of the “pathognomonic” findings of the NMT Metrics report for a dissociative-dominant individual. Other potential explanations for this finding include the fact that, given the lack of correlation at the bivariate level (Figure 1) between ELS and stress during childhood, children with severe stress during childhood may have had less ELS, setting them on a better developmental trajectory than children who experienced severe early, but not later, stress. In addition, we do not yet know how long it takes for a developmental insult to affect a developmental function. Finally, we know that neural systems organize to promote survival and resilience in the face of severe stress and adversity (Perry et al., 1995). Children who are currently living in highly chaotic, stressful and traumatic environments may be increasing, for example, their perceptual reasoning skills (Viezel et al., 2015) to contend with environmental stressors. We look forward to investigating this finding further.

## Limitations

Limitations of this dataset are detailed in previous publications (e.g., Hambrick et al., 2018) and also deserve mention here. The use of retrospective methods for obtaining developmental histories when studying how stress and trauma influences functioning is a common but debated practice (Greenhoot, 2013) given that retrospective child and/or caregiver reports can differ from actuarial reports (Hambrick et al., 2014) and prospective reports (Naicker et al., 2017). Yet, actuarial reports can also be problematic, because to be accurate, such reports rely on a timely and forthcoming report of a negative experience to an adult or authority figure, and may not contain the full picture of a child’s potentially traumatic experiences (Hambrick et al., 2014). In addition, some types of retrospective reporting have been found to show consistency over time, including maternal reports of child exposure to trauma around the time of pregnancy (Cammack et al., 2016; Wielaard et al., 2018). We also believe that the extensive training clinicians receive during NMT certification in how to complete the NMT Metrics mitigates some bias associated with the use of retrospective methods to assess children’s developmental histories. For example, clinicians are instructed to provide neutral “risk” scores when developmental history is unknown and receive in-depth training in how to interpret and access important corroborating information. Indeed, the use of child, caregiver, and caseworker report (when applicable) as well as available actuarial or “supplemental” information is recommended when seeking to document the clearest picture of a child’s developmental history (Dargis et al., 2018).

Regarding the rationale for conducting the factor analysis, it is worth noting that metric raters do have a general awareness that the NMT was developed to assess four broad domains of function (self-regulation, cognition, sensory integration, and relational). However, clinicians are not specifically trained to have an awareness of which Part C items are proposed to comprise which domain of function. Instead, clinicians are trained in how to complete each Part C item as if it is its own entity. In fact, providers do not receive a list of items hypothesized to comprise each domain in an effort to help preserve individualized rater attention to each Part C item. Although seasoned metric users may begin to infer which items are likely to represent, for example, cognitive vs. self-regulatory function, the metric rating process focuses on treating each item as unique. Moreover, an emphasis of NMT training is heterogeneity in functioning or helping clinicians recognize that children’s skills and abilities can be highly variable across brain-related functions.

Another study limitation was the monomethod, monoreporter (clinician) design, which may have caused inflated correlations (Spector, 2006). Our use of ridge regularization in tandem with cross-validation is an effective method for addressing multicollinearity to learn generalizable and stable estimates and was used to help manage this issue. Yet, as a reviewer correctly pointed out in the peer review process, the findings presented should be interpreted in light of the fact that the Part A and Part B indicators are correlated such that the presence or absence of one indicator may ultimately be related to the associations we find. From a clinical perspective, relational poverty in early childhood is a significant stressor. Our statistical modeling choices, however, were made in an effort to distinguish the effects of the Part A and B indicators on the factors in spite of their positive correlation.

An additional limitation of this study is related to the age categories in the current NMT metric. The age range in these categories are too broad to provide optimal examination of the timing of early experiences and potential associated outcomes during development. These associations may well shift during development and similar factor analysis and correlations for all of the age categories (not just the 8–10-year-old group) should be conducted. At present adequate numbers in all age categories for these analyses have not yet been reached in the NMT dataset. We are hopeful that future use of a modified early childhood version of the NMT metric will allow more granulation examination of potential relationships between early childhood experiences and functional outcomes at different points in development.

## Future Directions

We look forward to growing this dataset to allow for greater specification of how the timing of stress experiences influences specific aspects of neurodevelopmental function. We plan longitudinal follow-ups and the addition of a “research module” to append to the NMT Metric process. This research module will prompt clinicians to provide additional data regarding the children they serve, such as data from psychological testing and medication use. To understand the cascading effects of ELS, we must be able to observe which functions, when compromised, may lead to later compromise in other brain-related functional domains. Thus, the work will continually seek to adhere to a Research Domain Criteria-informed methodology in that we will “explore basic dimensions of functioning that span the full range of human behavior from normal to abnormal” when investigating how early life experience influences functioning (NIMH, n.d.). An early childhood version of the NMT metric and certification process is being developed. This version (NMT-EC) will focus on conception to age 4 and include 12 age categories (age ranges) over this first 4 years. We are hopeful that future use of a modified early childhood version of the NMT metric will allow more granulated examination of potential relationships between early childhood experiences and functional outcomes at different points in development.

In addition, we must better specify environmental variables influencing outcomes. For example, we need to begin collecting information regarding when or if children become child welfare-involved and/or removed from their homes, and when or if children and families begin receiving supportive services. In addition, identifying a subset of children in the dataset whose intrauterine experiences were reported with “high confidence” by clinicians will be key so that we can better understand the role of even ELS and relational buffers on neurodevelopmental outcomes.

## Conclusions

Current findings provide two key messages. The first is that the neurodevelopmental effects of ELS, which are typically better specified in basic as opposed to applied research (e.g., Bedrosian et al., 2018), are palpable in clinical settings. Understanding a patient’s very early experiences may indeed be important for promoting clinical improvement. The second is that not all ELS affects development in the same way. To improve “precision medicine” for children with developmental trauma, it is important to better understand how very early life experiences affect outcomes. Although interventions for trauma-exposed youth have proliferated and improved in recent decades, clinicians and researchers alike recognize that outcomes for children with severe ELS, especially when stress persists throughout development, are not consistently positive (e.g., Cakir et al., 2016) nor strong in effect (Fraser et al., 2013). These patients are some of the most perplexing clinical cases observed, sometimes presenting with severe dissociative responses, complex medical conditions, and sensory and self-regulatory impairments that tax traditional mental health delivery systems. Continued collaboration between basic and applied researchers regarding “brain programming by ELS” will be key to tackling the public health crisis of developmental trauma.

## Data Availability

The datasets generated for this study are available on request to the corresponding author.

## Author Contributions

EH participated in data analysis, conceptualization, and drafting the full manuscript. TB participated in data cleaning and analysis and drafting the “Materials and Methods” and “Results” sections. BP developed the data collection protocol, consulted on the analytic strategy, and participated in conceptualization. All authors contributed to manuscript revision, read and approved the submitted version.

## Conflict of Interest Statement

The authors declare that the research was conducted in the absence of any commercial or financial relationships that could be construed as a potential conflict of interest.
